# Toll-like receptor 7 stimulates production of specialized pro-resolving lipid mediators and promotes resolution of airway inflammation

**DOI:** 10.1002/emmm.201201891

**Published:** 2013-04-15

**Authors:** Ourania Koltsida, Sergey Karamnov, Katerina Pyrillou, Thad Vickery, Aikaterini-Dimitra Chairakaki, Constantin Tamvakopoulos, Paschalis Sideras, Charles N Serhan, Evangelos Andreakos

**Affiliations:** 1 Division of Immunogenetics, Center for Immunology and Transplantation, Biomedical Research Foundation Academy of AthensAthens, Greece; 2 Department of Anesthesiology, Perioperative, and Pain Medicine, Center for Experimental Therapeutics and Reperfusion Injury, Harvard Institutes of Medicine, Brigham and Women's Hospital, and Harvard Medical SchoolBoston, MA, USA; 3 Division of Pharmacology-Pharmacotechnology, Center for Basic Research, Biomedical Research Foundation Academy of AthensAthens, Greece

**Keywords:** airway inflammation, polyunsaturated fatty acid, resolution of inflammation, specialized pro-resolving mediator, Toll-like receptor

## Abstract

Although specialized pro-resolving mediators (SPMs) biosynthesized from polyunsaturated fatty acids are critical for the resolution of acute inflammation, the molecules and pathways that induce their production remain elusive. Here, we show that TLR7, a receptor recognizing viral ssRNA and damaged self-RNA, mobilizes the docosahexaenoic acid (DHA)-derived biosynthetic pathways that lead to the generation of D-series SPMs. In mouse macrophages and human monocytes, TLR7 activation triggered production of DHA-derived monohydroxy metabolome markers and generation of protectin D1 (PD1) and resolvin D1 (RvD1). In mouse allergic airway inflammation, TLR7 activation enhanced production of DHA-derived SPMs including PD1 and accelerated the catabasis of Th2-mediated inflammation. D-series SPMs were critical for TLR7-mediated resolution of airway inflammation as this effect was lost in *Alox15*^*−/−*^ mice, while resolution was enhanced after local administration of PD1 or RvD1. Together, our findings reveal a new previously unsuspected role of TLR7 in the generation of D-series SPMs and the resolution of allergic airway inflammation. They also identify TLR stimulation as a new approach to drive SPMs and resolution of inflammatory diseases.

## INTRODUCTION

Prolonged or non-resolving inflammation underlies the pathogenesis of a wide range of virtually unrelated diseases ranging from autoimmune, metabolic and neurological disorders to allergies (Medzhitov, 2008; Nathan & Ding, 2010). Despite that, mechanisms that control resolution of inflammation are not well understood, mainly because resolution was envisaged for a long time to occur passively following the decline of pro-inflammatory responses. However, this paradigm has recently changed. It has become apparent that resolution or catabasis (Bannenberg et al, 2005) of inflammation is an active and tightly regulated process that brings about the catabolism of pro-inflammatory mediators, the removal of inflammatory cells and the restitution of the tissue in a timely and highly coordinated manner (Nathan & Ding, 2010; Serhan et al, 2007). This is critically dependent on the activation of biochemical programs involving oxygenated lipids derived from polyunsaturated fatty acids, altogether termed specialized pro-resolving lipid mediators (SPMs) (Gilroy et al, 2004; Serhan et al, 2008). SPMs are biosynthesized in inflammatory exudates via the consecutive actions of lipoxygenase (ALOX-5, ALOX-12 and ALOX-15) and/or cyclooxygenase (COX-2) enzymes and include protectins and D-series resolvins derived from the omega-3 (ω-3) fatty acid docosahexaenoic acid (DHA), E-series resolvins derived from eicosapentanoic acid (EPA), and lipoxins derived from omega-6 fatty acids (Serhan et al, 2008). Of interest, enzymes that are essential for the production of SPMs such as COX-2 and ALOX-5 are also the targets of major non-steroidal anti-inflammatory drugs (*e.g.* ibuprofen, naproxen, celecoxib) or anti-leukotriene treatments (*e.g.* zileuton), raising the possibility that certain widely used anti-inflammatory drugs may actually inhibit/delay resolution of inflammation despite their potent anti-inflammatory effects (Bhavsar et al, 2010; Serhan et al, 2008).

Allergic asthma is a chronic inflammatory disease of the airways characterized by Th2-mediated immune responses to common aeroallergens in genetically susceptible individuals (Holgate & Polosa, 2008; Kim et al, 2010). Th2 responses most frequently develop early in life, persist even during asymptomatic periods and are exacerbated in response to an environmental trigger such as allergen exposure, viral infection or other irritants leading to recurrent episodes of wheezing, breathlessness and coughing (altogether described as an asthmatic attack), and eventually the progressive decline of lung function. Although the intensity and duration of Th2 exacerbations determine the severity of the asthmatic attack (Bousquet et al, 2000; Jackson et al, 2011; Locksley, 2010), the molecular mechanisms and pathways controlling persistence or resolution of Th2 allergic responses in the airways are largely unknown, while therapies that actively resolve aberrant immune responses in asthma have yet to be identified.

Multiple factors are proposed to influence the development or persistence of inflammation in allergic asthma. Among them, Toll-like receptors (TLRs) have taken centre stage by virtue of their ability to recognize common allergens, microbial structures and endogenous ‘danger’ signals, and promote pro-inflammatory responses in the airways (Edwards et al, 2012; Palm et al, 2012). For instance, TLR4 senses house dust mite and ragweed pollen (Hammad et al, 2009; Li et al, 2011; Trompette et al, 2009), possibly through molecular mimicry, while TLR3 senses viral double stranded RNA, and both contribute to the initiation or exacerbation of inflammation (Jeon et al, 2007; Reuter et al, 2012; Torres et al, 2010). However, not all TLRs are detrimental. TLR7 and TLR8 that recognize single stranded RNA of respiratory viruses, and TLR9 that recognizes unmethylated CpG islands of viral or bacterial DNA, exhibit surprising immunoregulatory/immunomodulatory activity (Hennessy et al, 2010; Kanzler et al, 2007). In mouse models of allergic airway disease, prophylactic administration of TLR7/8 and TLR9 agonists prevents the development of airway inflammation and hyperresponsiveness, through mechanisms that extend beyond the modulation of the Th1/Th2 cytokine balance (Fonseca & Kline, 2009; Moisan et al, 2006; Quarcoo et al, 2004; Sel et al, 2007; Xirakia et al, 2010). Moreover, in proof-of-concept clinical trials in humans, treatment with TLR7, TLR8 or TLR9 agonists reduces symptoms of allergic diseases such as asthma (Cytos Biotechnology AG. Placebo-controlled phase II study shows CYT003-QbG10 is safe and efficacious for the treatment of allergic asthma. http://www.cytos.ch/?id=1572), rhinitis (Horak, 2011) or rhinoconjunctivitis (Klimek et al, 2011), yet the mechanistic basis for these effects is lacking. In this study, we used an established mouse model of acute allergic airway inflammation, relevant to human allergic asthma, to address the role of TLR7 in the resolution of inflammation and identify specific SPM networks involved.

## RESULTS

### Spatiotemporal resolution of allergic airway inflammation in mice after discontinuation of allergen challenge

**Figure 1:**
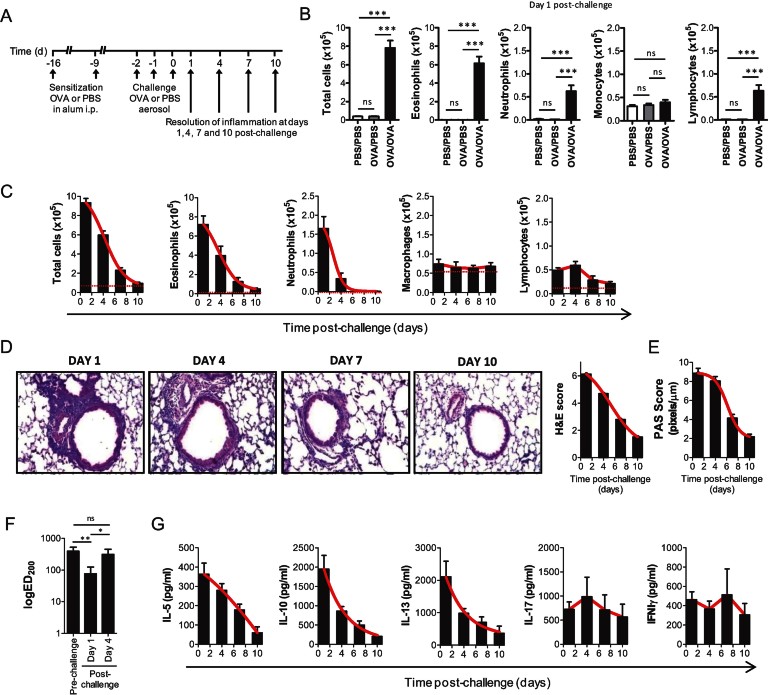
Spatiotemporal analysis of resolution of allergic airway inflammation post-OVA challenge. A. Protocol of induction and time-course of resolution of allergic airway inflammation post-allergen challenge in wild-type mice. B. Total cell counts in the BALF of PBS sensitized and challenged (PBS/PBS, *n* = 10), OVA sensitized and PBS challenged (OVA/PBS, *n* = 10) or OVA sensitized and challenged (OVA/OVA, *n* = 30) mice at Day 1 post-challenge. Results are expressed as mean ± SEM of 10–30 mice per group from four independent experiments. C. Total and differential cell counts in the BALF of OVA sensitized and challenged mice (OVA/OVA) at Days 1, 4, 7 and 10 post-allergen challenge expressed as mean ± SEM of 30 mice per group from four independent experiments. Red dotted lines: cell counts of healthy (unchallenged) controls. D. Histological assessment of lung inflammation of OVA sensitized and challenged (OVA/OVA) mice at Days 1, 4, 7 and 10 post-allergen challenge. Haematoxylin and eosin (H&E)-stained lung sections and histological scoring expressed as mean values ± SEM of 10 mice/group from two independent experiments are shown. E. Histological assessment of mucus secretion of OVA sensitized and challenged (OVA/OVA) mice at Days 1, 4, 7 and 10 post-allergen challenge. Morphometric analysis of Periodic acid Schiff (PAS)-stained sections expressed as mean values ± SEM of 10 mice/group from two independent experiments are shown. F. Airway hyperesponsiveness of mice before (pre-challenge) and at Days 1 and 4 post-challenge. LogED_200_, the effective dose of MCh required to increase mean lung resistance by 200% from baseline, is shown. Values represent the mean ± SEM of seven mice per group. One representative from two independent experiments. G. Allergen-specific effector T cell responses in mediastinal LN cultures of OVA sensitized and challenged (OVA/OVA) mice at Day 1, 4, 7 and 10 post-allergen challenge. Cytokine levels are expressed as mean values ± SEM in supernatants of OVA-stimulated mediastinal LN cultures of 10 mice per group from two independent experiments. *p < 0.05, **p < 0.01, ***p < 0.001, ns: non-significant compared to vehicle-treated control.

Although mouse models of asthma based on ovalbumin (OVA) sensitization and challenge are widely used to study immuno-inflammatory disease mechanisms, little information exists about the spatiotemporal resolution of inflammation after discontinuation of OVA challenge. We therefore monitored the kinetics of resolution of various inflammatory and disease-related parameters in a systematic manner (Fig 1A). Upon three consecutive OVA challenges, OVA sensitized mice developed a strong eosinophilic inflammation in the airways that was absent from PBS challenged mice or healthy controls (Fig 1B). Inflammation peaked at Day 1 post-challenge and declined to almost homeostatic levels by Day 10. Total cell counts in bronchoalveolar lavage fluid (BALF) decreased by 36% by Day 4, 75% by Day 7 and >90% by Day 10 to levels that were not statistically different to healthy controls (Fig 1C). These kinetics were closely followed by eosinophils (34% by Day 4, 83% by Day 7 and >90% by Day 10) which also constitute the main infiltrating cells in the BALF. In contrast, neutrophil numbers decreased by 80% by Day 4 and 100% by Day 7, lymphocytes by 32% by Day 7 and 57% by Day 10 (Fig 1C). At the tissue level, inflammatory cell infiltrates peaked at Day 1 post-challenge and cleared with somewhat slower kinetics than total BALF cells (Fig 1D) as did goblet cell metaplasia, a consequence of allergic airway inflammation. Thus, although inflammatory cell infiltrates and goblet cells in the lung gradually declined, they remained at significant levels even at Day 10 post-challenge (Fig 1E). On the contrary, airway hyper-responsiveness, another consequence of allergic airway inflammation and the cardinal feature of human asthma, was detectable at Day 1 post-challenge, as indicated by the much lower effective dose of methacholine (MCh) required to increase mean lung resistance 200% from baseline (ED_200_), but not at Day 4 post-challenge or thereafter (Fig 1F and Supporting Information Fig S1). Finally, OVA-specific Th2 cell responses peaked at Day 1 post-challenge and gradually regressed to pre-challenge levels by Day 10 (Fig 1G). Thus, despite some temporal differences in the resolution of distinct inflammatory parameters, overall inflammation peaks at Day 1 post-challenge and gradually declines thereafter. This makes this model a potent setup for studying resolution of an acute allergic inflammatory response.

### TLR7 regulates resolution of allergic airway inflammation in mice

**Figure 2:**
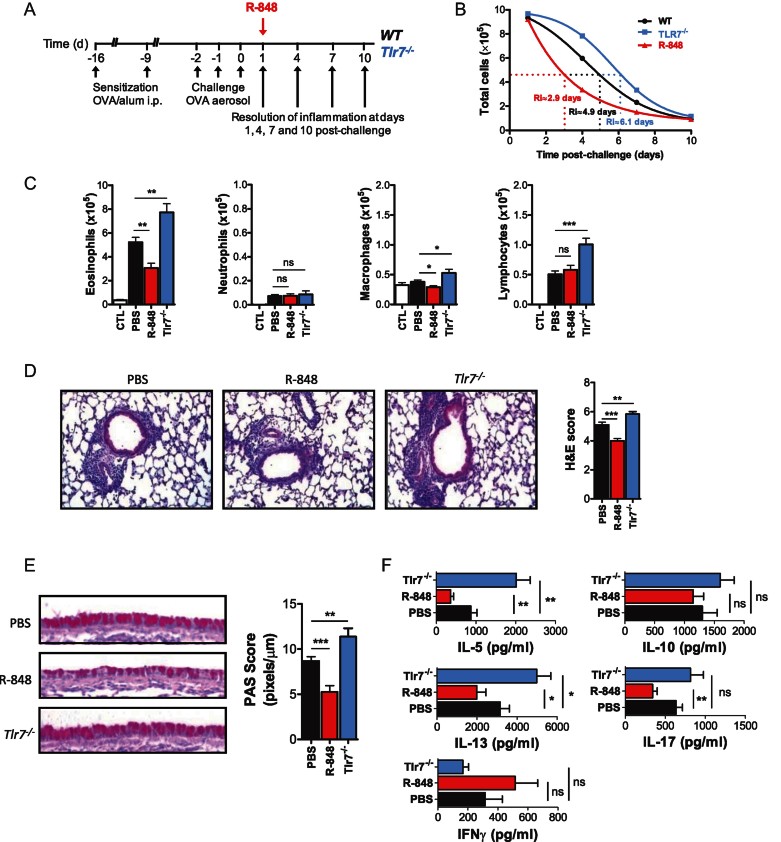
TLR7 promotes resolution of allergic airway inflammation in mice. A. Protocol of R-848 (200 µg/mouse) treatment in wild-type mice, and comparison of resolution in wild-type and *Tlr7*^*−/−*^ mice. B. Total cell counts in the BALF of OVA sensitized and challenged (OVA/OVA) wild-type (*n* = 30), R-848-treated (*n* = 30) and *Tlr7*^*−/−*^ (*n* = 20) mice at Days 1, 4, 7 and 10 post-challenge expressed as mean ± SEM of 20–30 mice per group. C. Differential cell counts in BALF of wild-type (*n* = 30), R-848-treated (*n* = 30) and *Tlr7*^*−/−*^ (*n* = 20) mice at Day 4 post-challenge expressed as mean ± SEM of 20–30 mice per group are shown. D. Histological assessment of lung inflammation of wild-type (*n* = 18), R-848-treated (*n* = 18) and *Tlr7*^*−/−*^ (*n* = 10) mice at Day 4 post-challenge. Haematoxylin and eosin (H&E)-stained lung sections and histological scoring expressed as mean values ± SEM from 10 to 18 mice/group are shown. E. Histological assessment of mucus secretion of wild-type (*n* = 18), R-848-treated (*n* = 18) and *Tlr7*^*−/−*^ (*n* = 10) mice at Day 4 post-challenge. Periodic acid Schiff (PAS)-stained sections and morphometric analysis expressed as mean values ± SEM from 10 to 18 mice/group are shown. F. Allergen-specific effector T cell responses in mediastinal LN cultures of OVA/OVA wild-type (*n* = 18), R-848-treated (*n* = 18) and *Tlr7*^*−/−*^ (*n* = 10) mice at Day 4 post-challenge. Cytokine levels are expressed as mean values ± SEM in supernatants of OVA-stimulated mediastinal LN cultures of 10–18 mice per group. *p < 0.05, **p < 0.01, ***p < 0.001, ns: non-significant compared to vehicle (PBS)-treated control.

Using this model, we next evaluated the hypothesis that TLR7 modulates resolution of allergic airway inflammation (Fig 2A). Surprisingly, we found that *Tlr7*^*−/−*^ mice exhibited significantly delayed resolution of inflammation. At Day 4 post-challenge, total inflammatory cells and eosinophils in the BALF of *Tlr7*^*−/−*^ mice were ∼31 and ∼48% higher than in wild-type controls, respectively (Fig 2B and C). This was associated with increased inflammatory cell infiltrates in the lung, more pronounced goblet cell metaplasia in the airways and higher Th2 cell responses in lung-draining lymph nodes (Fig 2D–F). In contrast, wild-type mice treated with R-848, a specific TLR7 agonist in mice, exhibited accelerated resolution of allergic airway inflammation. R-848 treatment reduced the number of inflammatory cells and eosinophils in the BALF by ∼37 and ∼35%, respectively (Fig 2B and C), decreased the presence of inflammatory cell infiltrates and goblet cells in the lung (Fig 2D and E), and down-regulated allergen-specific Th2 responses in lung-draining lymph nodes (Fig 2F). These effects were completely abrogated when R-848 was administered to *Tlr7*^*−/−*^ mice (Supporting Information Fig S2), indicating the specificity of the response. Examination of the resolution interval (*R*_i_; defined as the time required for cell numbers to reach 50% of the numbers observed at the peak of inflammation (Bannenberg et al, 2005)) of total inflammatory cells in the BALF revealed a shift from 4.9 days for wild-type mice to 2.9 days for R-848-treated mice and 6.1 days for *Tlr7*^*−/−*^ mice (Fig 2B). Taken together, these results suggest that TLR7 activation promotes resolution of inflammation in experimental asthma.

### TLR7 activation drives the production of DHA-derived SPMs in the lung

**Figure 3:**
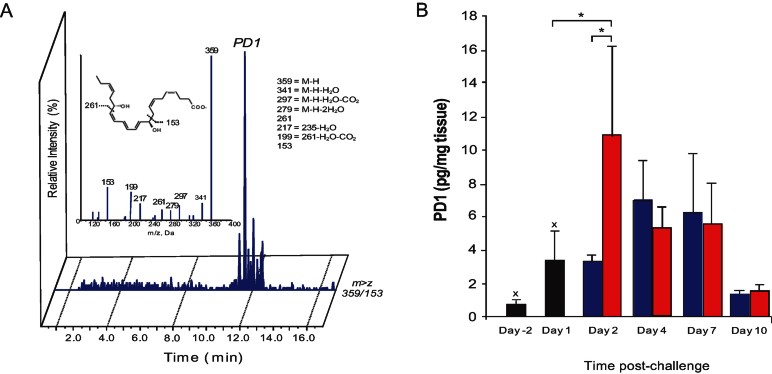
R-848 stimulates production of SPMs in murine allergic airway inflammation. OVA sensitized and challenged mice treated with 200 µg R-848 or vehicle control (PBS) on Day 1 post-challenge were sacrificed at target time points and lungs analyzed. A. LC-MS–MS chromatogram of Protectin D1 (PD1) identified in R-848-treated mouse lung. B. Time-course of PD1 production during murine allergic airway inflammation. Black bars indicate baseline levels before administration of R-848. Blue bars indicate vehicle (PBS). Red bars indicate treatment with R-848. *p < 0.05; ^X^*p* < 0.05 compared to 0.

In view of the potent pro-resolving effects of TLR7 activation in murine allergic airway inflammation, we compared the lipidomic profiles of lungs from OVA challenged mice treated with vehicle or R-848 using novel liquid chromatography-tandem mass spectroscopy mediator lipidomics (LC-MS/MS) (Yang et al, 2011). We identified the production of monohydroxy biomarkers from the DHA, arachidonic acid (AA), and EPA metabolomes, and the generation of AA-derived prostaglandins and leukotrienes (see Supporting Information Table S1 and Fig S3). These were increased in OVA challenged mice at Day 1 post-challenge compared to unchallenged (non-inflamed) controls, and exhibited a variable course during the resolution of airway inflammation. Of particular interest, we identified protectin D1 (PD1/NPD1), a downstream bioactive product from DHA and a potent pro-resolving lipid mediator, present at all time points examined. PD1/NPD1 production was gradually increased in resolving inflammation, and only declined at Day 10 post-challenge once inflammation had resolved (Fig 3A and B). We also identified both 17-hydroxydocosahexaenoic acid (17-HDHA) and 14-hydroxydocosahexaenoic acid (14-HDHA), biosynthetic pathways markers for PD1 and maresin 1. These were elevated at the peak of the inflammatory response and increased further as resolution progressed before declining at Day 10 (Supporting Information Table S1). Notably, PD1 levels, as well as 17-HDHA and 14-HDHA, were rapidly up-regulated upon R-848 treatment, suggesting that conversion of endogenous DHA to its bioactive products may be central to TLR7-mediated resolution of airway inflammation.

### TLR7 activation initiates SPM biosynthesis in mouse and human cells

**Figure 4:**
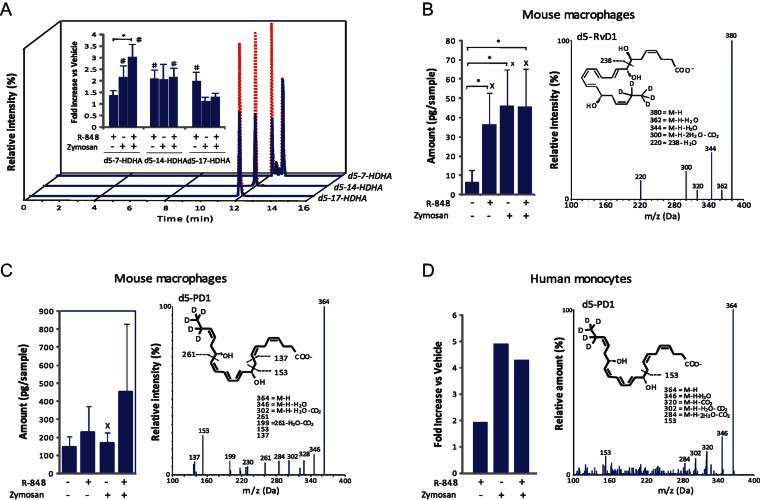
R-848 stimulates production of SPMs and their precursors from deuterium-labelled DHA in monocytes and macrophages. A. LC-MS-MS chromatograms of d5-7-HDHA, d5-14-HDHA and d5-17-HDHA produced by untreated (solid blue line) and R-848-treated (dashed red line) mouse macrophages. B,C. Increases in mouse macrophage d5-RvD1 and d5-PD1: quantitation (left) and LC-MS-MS identification (right) by R-848-treated mouse macrophages. Results are expressed as mean ± SEM of *n* = 4 for d5-RvD1 and *n* = 3 for d5-PD1. D. Increase in human monocyte d5-PD1: quantitation (left) and LC-MS–MS identification (right) by R848-treated human monocytes. Results are representative of *n* = 3. Proresolving lipid mediators were identified according to published criteria (Yang et al, 2011). *p < 0.05; ^X^*p* < 0.05 compared to 0; ^#^*p* < 0.05 compared to 1.

**Figure 5:**
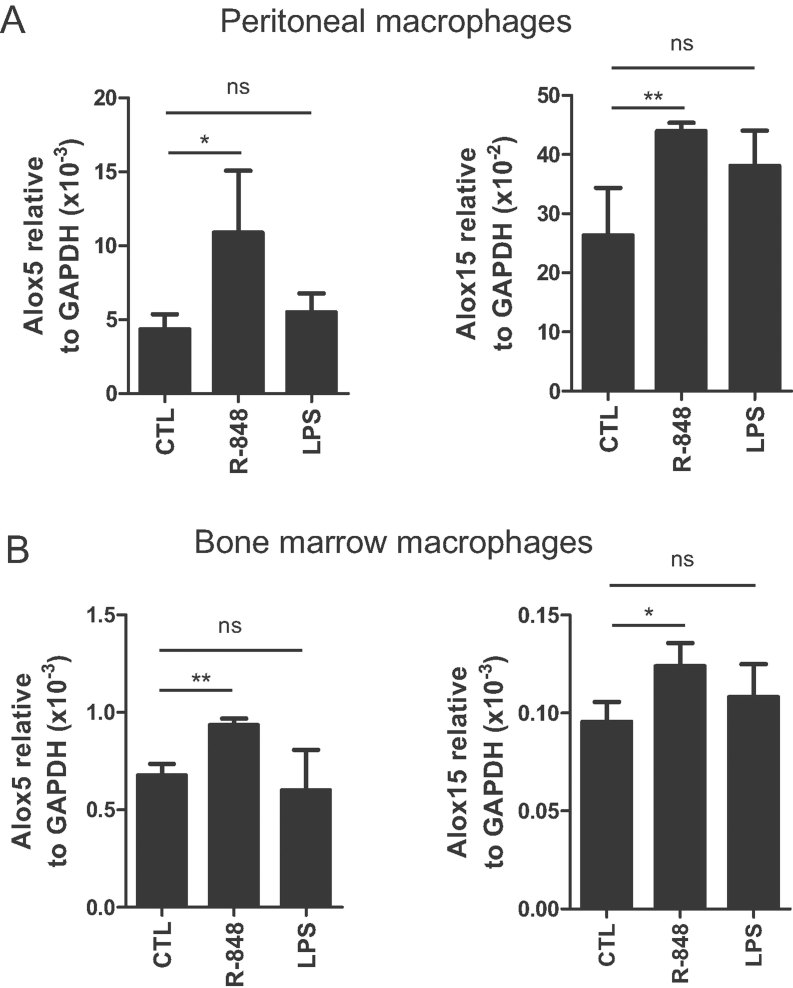
R-848 up-regulates the expression of 5-lipoxygenase and 12/15-lipoxygenase enzymes. Mouse macrophages were stimulated for 1 h with 5 µg/ml R-848, 1 µg/ml LPS or vehicle-treated control (CTL) and analyzed by real-time PCR. A. Alox-5 and Alox-15 mRNA levels in peritoneal macrophages expressed as mean levels ± SEM relative to GAPDH (*n* = 6 from two independent experiments). B. Alox-5 and Alox-15 mRNA levels in bone marrow-derived macrophages expressed as mean levels ± SEM relative to GAPDH (*n* = 6–10 from two independent experiments). *p < 0.05, **p < 0.01, ns: non-significant compared to CTL.

Circulating substrates, polyunsaturated fatty acids DHA and EPA and their flux into inflammatory milieu is a known essential source of SPMs biosynthesis and as a result, an important step in resolution of inflammation (Kasuga et al, 2008). To determine whether TLR7 (TLR7/8 in humans) activation directly induces the production of SPMs, we used d5-containing DHA and tracked deuterium incorporation into SPM profiles biosynthesized by mouse and human cells using systematic LC-MS/MS-based mediator lipidomics. We found significant increases in 7-hydroxydocosahexaenoic acid (d5-7-HDHA), d5-14-HDHA and d5-17-HDHA (Fig 4A) as well as in SPMs d5-Resolvin D1 (RvD1; Fig 4B) production by R-848-treated mouse macrophages when compared to cells exposed to vehicle alone. PD1 was also produced but was not increased further after stimulation (Fig 4C). Similarly, we found significant increases in monohydroxy-containing products (Supporting Information Fig S4) and d5-PD1 production in R-848-treated human monocytes (Fig 4D). The presence of deuterium in the structure of RvD1 and PD1 confirmed that TLR7 receptor activation enhanced DHA utilization and biosynthesis of specialized pro-resolving lipid mediators by both murine and human cells. Notably, TLR7 but not TLR4 stimulation also up-regulated in macrophages the expression of 5-lipoxygenase and/or 12/15-lipoxygenase enzymes (Fig 5), rate-limiting components in the metabolism of DHA to its bioactive derivatives (Hong et al, 2003; Miyata et al, 2013; Serhan et al, 2006). These data suggest a direct role of the TLR7 pathway in the generation of SPMs which can be explained, in part, by its ability to increase the expression of lipoxygenase enzymes.

### Endogenous production of D-series SPMs is critical for TLR7-mediated resolution of inflammation

**Figure 6:**
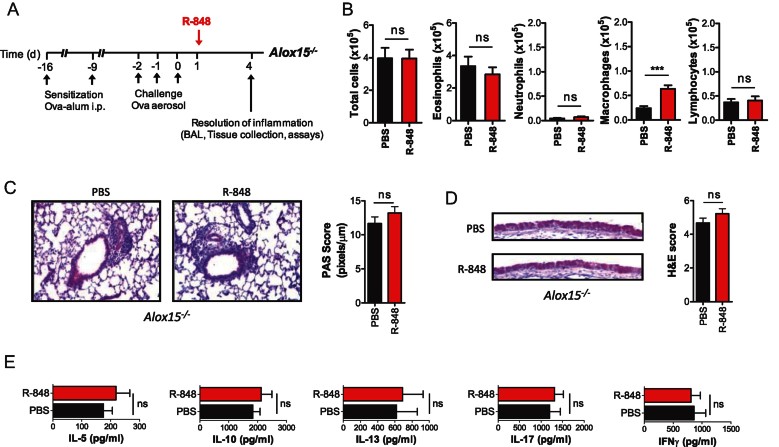
12/15-lipoxygenase and production of D-series SPMs are essential for R-848-mediated resolution of allergic airway inflammation. A. Protocol of R-848 (200 µg/mouse) or vehicle (PBS) administration in *Alox15*^*−/−*^ mice. B. Total and differential cell counts in BALF of OVA sensitized and challenged (OVA/OVA) mice at Day 4 post-challenge. Results are expressed as mean ± SEM of 8 mice per group. C. Histological assessment of lung inflammation of *Alox15*^*−/−*^ mice at Day 4 post-challenge. Haematoxylin and eosin (H&E)-stained lung sections and histological scoring expressed as mean values ± SEM from 8 mice/group are shown. D. Histological assessment of mucus secretion of *Alox15*^*−/−*^ mice at Day 4 post-challenge. Periodic acid Schiff (PAS)-stained sections and morphometric analysis expressed as mean values ± SEM from 8 mice/group are shown. E. Allergen-specific effector T cell responses in mediastinal LNs of *Alox15*^*−/−*^ mice at Day 4 post-challenge. Cytokine levels are expressed as mean values ± SEM in supernatants of OVA-stimulated mediastinal LN cultures of 8 mice per group. ns: non-significant compared to vehicle-treated control.

To evaluate whether endogenous SPM production is ‘rate-limiting’ in TLR7-mediated resolution of allergic airway inflammation, we employed *Alox15*^*−/−*^ mice. These animals lack 12/15-lipoxygenase and thus do not convert DHA to its bioactive components PD1 and RvD1 (Gronert et al, 2005; Yamada et al, 2011). We found that in these lipoxygenase-deficient mice the beneficial effects of R-848 treatment were abrogated. Airway inflammation assessed by determining inflammatory cell numbers in BALF and leukocytic cell infiltration did not differ significantly between R-848-treated and control mice (Fig 6A–C). Goblet cell metaplasia in the airways (Fig 6D) and allergen-specific Th2 responses in lung-draining lymph nodes (Fig 6E) were also not different between these two groups. These findings suggest that R-848-mediated pro-resolving activity is directly linked to lipoxygenase-dependent production of D-series SPMs such as PD1 and RvD1 *in vivo*.

### Intranasal administration of the D-series SPMs PD1 and RvD1 promotes resolution of established allergic airway inflammation

**Figure 7:**
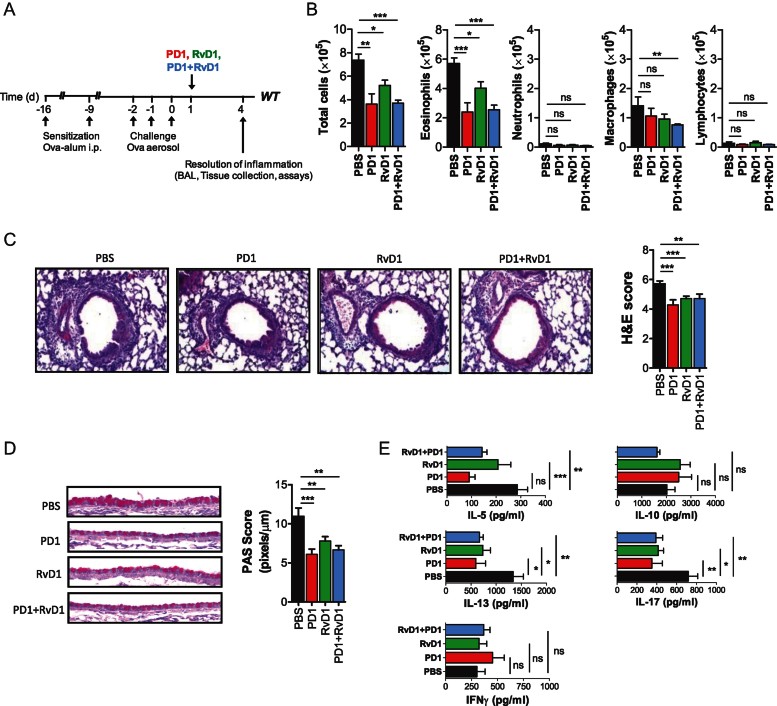
Intranasal administration of PD1 or RvD1 promotes resolution of allergic airway inflammation. A. Protocol of PD1, RvD1, PD1 and RvD1 (100 ng/mouse) or vehicle (PBS) administration in wild-type mice. B. Total and differential cell counts in BALF of OVA sensitized and challenged (OVA/OVA) mice at Day 4 post-challenge. Results are expressed as mean ± SEM of 9 mice per group. C. Histological assessment of lung inflammation at Day 4 post-challenge. Haematoxylin and eosin (H&E)-stained lung sections and histological scoring expressed as mean values ± SEM from 9 mice/group are shown. D. Histological assessment of mucus secretion at Day 4 post-challenge. Periodic acid Schiff (PAS)-stained sections and morphometric analysis expressed as mean values ± SEM from 9 mice/group are shown. E. Allergen-specific effector T cell responses in mediastinal LNs at Day 4 post-challenge. Cytokine levels are expressed as mean values ± SEM in supernatants of OVA-stimulated mediastinal LN cultures of 9 mice per group. *p < 0.05, **p < 0.01, ***p < .001, ns: non-significant compared to vehicle-treated control.

Finally, we determined the impact of PD1 or RvD1 treatment to the resolution of airway inflammation in wild-type mice. We observed that a single intranasal administration of PD1 or RvD1 given at physiologic range, that is nanograms per mouse, at Day 1 post-challenge effectively accelerated the catabasis of airway inflammation. Total inflammatory cell and eosinophil numbers in the BALF were both reduced in PD1 and RvD1-treated animals compared to vehicle treated controls (Fig 7A and B). Inflammatory cell infiltrates in the lung, goblet cell metaplasia in the airways and Th2 cell responses in lung-draining lymph nodes were also similarly reduced (Fig 7C–E). However, neither synergistic nor additive effects between PD1 and RvD1 were observed. These findings demonstrate that D-series SPMs such as PD1 and RvD1 exhibit potent pro-resolving activity in mice with already established allergic airway inflammation.

## DISCUSSION

Here we report that TLR7, a receptor recognizing viral ssRNA and self-RNA released during tissue injury or necrosis, mobilizes the ω-3 DHA-derived biosynthetic pathways leading to the generation of D-series SPMs. Using targeted lipidomics, we show that in mouse peritoneal macrophages and human peripheral blood monocytes, TLR7 drives the production of DHA-derived monohydroxy metabolites including d5-14-HDHA and d5-17-HDHA, and the generation of PD1 as well as RvD1. Using a mouse model of allergic airway inflammation, we further show that TLR7 enhances the generation of DHA-derived SPMs including PD1 *in vivo*. Finally, we demonstrate that TLR7-mediated induction of DHA-derived SPMs is functionally important for the catabasis of Th2-mediated inflammation, as this effect is lost in *Alox15*^*−/−*^ mice, while resolution is enhanced after local administration of PD1 or RvD1. These findings link for the first time the activation of TLR7, or any TLR in general, with the production of resolvins and protectins, and the resolution of inflammation. They also extend our current view on the mechanism of action of TLR7 agonists and suggest that these molecules act at multiple levels to counter-regulate allergic airway disease. Through the induction of type I IFNs (Xirakia et al, 2010), they rapidly shut down Th2-mediated pro-inflammatory responses in the airways. Through the production of D-series SPMs, they promote resolution of allergic airway inflammation. Finally, through the generation of regulatory T cell populations secreting IFN-γ (Sel et al, 2007), they ensure long-term suppression of allergic responses and long-term remission of allergic airway disease.

Our present results may at first appear paradoxical. TLRs are major pattern recognition receptors involved in the initiation and propagation of inflammation, and the ability of TLR7 to activate a key pro-resolving pathway seems surprising. However, it is often overlooked that inflammation evolved as an adaptive response for restoring homeostasis, and that a successful inflammatory response is always followed by a resolution and repair phase (Medzhitov, 2008; Serhan et al, 2008). Accordingly, there is evidence that pro-resolving pathways are set in motion at the beginning of an inflammatory response, and become predominant as inflammation enters into the resolution phase (Serhan et al, 2008). In addition, there are reports that bacteria or yeast carbohydrates activate PUFA-derived lipid mediator networks, and although the precise mechanisms are not known, pattern recognition receptors are likely involved (Chiang et al, 2012; Kasuga et al, 2008). Finally, there are studies suggesting that PD1 and RvD1 are also part of the antimicrobial defence and enhance bacterial phagocytosis from macrophages and neutrophils without stimulating uncontrolled inflammation and collateral tissue damage (Chiang et al, 2012) or mediating immunosuppression (Norris & Dennis, 2012; Serhan, 2007; Serhan et al, 2008). Therefore, our findings are compatible with the known biology of TLRs in innate immunity, and suggest that induction of SPMs by TLR7 constitutes an important negative feedback loop that keeps inflammation under control and ensures its proper resolution.

A question that arises from our study is how DHA-derived SPMs mediate resolution of Th2-mediated allergic airway inflammation. Topical (intranasal) treatment of synthetic PD1 or RvD1 given at physiologic range post-allergen challenge accelerates resolution of established Th2 responses in the airways, which is in line with prophylactic effects of systemic PD1 administration on the development of Th2 responses in the lung previously described (Levy et al, 2007). This could be due to direct effects on Th2 cell activation and cytokine production, T cell migration or T cell apoptosis (Ariel et al, 2005). Alternatively, this could result from indirect effects of PD1 on macrophage phagocytic activity and non-inflammatory apoptotic cell clearance, de-activation of the endothelium, inhibition of transepithelial migration, or down-regulation of pro-inflammatory cytokines and enzymes, fostering overall resolution of inflammation without specifically targeting the Th2 response (Serhan, 2007; Serhan et al, 2008). In support of the former possibility, 12/15-lipoxygenase has been shown to be required for optimal expression of IFN-γ in other experimental settings (Dioszeghy et al, 2008; Middleton et al, 2006; Zhao et al, 2002). Similar mechanisms may also be employed by RvD1. Although much less is known about its role in Th2-mediated inflammation, RvD1 has been shown to inhibit leukotriene-induced secretions from conjunctival goblet cells (Dartt et al, 2011), suggesting that resolution of goblet cell metaplasia and mucous hypersecretion during allergic airway inflammation may be due to direct actions of SPMs in the lung. Therefore, SPMs such as PD1 and RvD1 stimulate resolution of inflammation via multi-level mechanisms that control the catabasis of inflammation and the restoration of tissue homeostasis.

Another important question is how TLR7 enhances the biosynthesis of DHA-derived SPMs. We found that in cultured peritoneal and bone marrow-derived macrophages TLR7 stimulation up-regulates the expression of 5-lipoxygenase and/or 12/15-lipoxygenase enzymes, rate-limiting components in the metabolism of DHA to its bioactive derivatives (Hong et al, 2003; Miyata et al, 2013; Serhan et al, 2006). Notably, 12/15-lipoxygenase expression has been shown to mark alternatively activated macrophages and to play a key role in the clearance of apoptotic cells and the maintenance of tissue homeostasis (Uderhardt et al, 2012), raising the possibility that TLR7 stimulation, alternatively activated macrophage function and DHA-derived SPM production are linked. However, TLR7-mediated regulation of lipoxygenases may not be the only mechanism involved as substrate availability is also critical. *In vivo*, regulation of substrate availability is complex and involves multiple sources including lung resident cells and infiltrating leukocytes that carry esterified DHA in membrane phospholipids (such as phosphatidylethanolamine and phosphatidylcholine), and oedema which provides unesterified DHA to local sites of inflammation (Hong et al, 2003; Kasuga et al, 2008; Marcheselli et al, 2003; Serhan, 2010). It is therefore possible that TLR7 enhances the biosynthesis of D-series SPMs by also affecting substrate availability as it has been previously shown that R-848 can mobilize eicosanoids from esterified lipids and increase oedema formation (Hattermann et al, 2007; Norris & Dennis, 2012; Takeuchi & Akira, 2010).

Other SPMs have also been studied in asthma. Resolvin E1 (RvE1) exhibits potent pro-resolving activity in allergic airway inflammation by inhibiting Th17 without affecting Th2 responses in the airways while increasing IFN-γ (Haworth et al, 2008). Lipoxin A_4_, biosynthesized from arachidonic acid following lipid mediator class switching, is another pivotal SPM. LXA_4_ is present in sputum and BALF of severe asthmatic patients (Levy et al, 2005; Planaguma et al, 2008) and has been identified in BALF of BALB/c mice with allergic airway inflammation (Haworth et al, 2008). Unfortunately, we have not examined BALF in our setting in C57BL/6 mice while we have not been able to detect LXA_4_ above the limit of quantification in lung tissue. LXA_4_ prevents the development of Th2 responses and Th2-mediated inflammation in the lung although its pro-resolving potential has not been sufficiently investigated (Levy et al, 2002). Hence, the description in our study of an endogenous mechanism that drives the catabasis of acute allergic airway inflammation, and the identification of a pharmaceutical agonist that can accelerate this process, constitute important steps forward towards understanding resolution in asthma.

A surprising observation in our studies was that *Tlr7*^*−/−*^ mice exhibited significantly delayed resolution of inflammation. This pointed to the presence of endogenous TLR7 ligand(s) and suggested that TLR7 is part of an endogenous regulatory mechanism that acts to keep inflammation under control and restore tissue homeostasis. In an effort to identify potential TLR7 ligands, we tested for the presence of extracellular RNA in inflamed lung using an established protocol (Ganguly et al, 2009; Salagianni et al, 2012) and found that RNA aggregates – presumably released from damaged cells – are detectable in acellular areas of the inflamed tissue (Supporting Information Fig S5). These are likely to constitute endogenous ligands for TLR7 and contribute to TLR7-mediated signalling *in vivo*. Further studies will be needed to define the exact nature and immunostimulatory potential of these structures as well as their cellular targets as multiple immune (alveolar macrophages, myeloid and plasmacytoid dendritic cells, eosinophils) and structural cells (bronchial epithelial cells) in the lung expressing abundant levels of TLR7 (Bessa et al, 2009; Cherfils-Vicini et al, 2010; Demedts et al, 2006; Maris et al, 2006; Nagase et al, 2003; Xirakia et al, 2010). Our present results have direct therapeutic implications for the treatment of aberrant inflammatory responses during acute exacerbations of asthma. Traditional approaches involve the administration of inhaled or systemic corticosteroids, often in combination with anti-leukotriene inhibitors, but their therapeutic efficacy is limited, especially in the most severe cases of the disease (Jackson et al, 2011). Rather than inhibiting molecular events that occur at the beginning of the immune response, a process that may thwart the body's own attempt to heal, our study now proposes a new approach for taming inflammation by directly stimulating endogenous resolution mechanisms; the induction of SPMs via the activation of the TLR7 pathway. TLR7 activation may be particularly suited for the treatment of acute exacerbations of asthma as these are often due to viral infections, and in addition to its potent pro-resolving function, TLR7 also exhibits strong antiviral activity (Blasius & Beutler, 2010; O'Neill & Bowie, 2010). As several TLR7 or TLR7/8 agonists are under clinical development for the treatment of various diseases including respiratory allergies (Hennessy et al, 2010; Kanzler et al, 2007), their efficacy in clinical trials of allergic asthma exacerbations are eagerly awaited.

In summary, our results reveal a new previously unanticipated role of TLR7 in the production of DHA-derived SPMs and the catabasis of Th2-mediated inflammation. They demonstrate that a TLR, and a selective TLR agonist, drive the production of SPMs and actively mediate resolution of inflammation. They also suggest the existence of a negative feedback loop triggered by TLR7 that counter-regulates inflammation and ensures its proper resolution and catabasis. This broadens our current view of TLR function in health and disease, and has profound implications for the development of TLR agonists and antagonists in the clinic. Moreover, our study unravels a new mode of action of resiquimod, an existing antiviral drug of the imidazoquinoline family, and establishes TLR7/8 agonists as the first pharmacological agents beyond aspirin and statins that are capable of directly inducing the lipoxygenase-dependent biosynthetic pathways leading to the production of SPMs. This strengthens the rationale for the application of TLR7/8 agonists for the treatment of allergic asthma and respiratory allergies in general, and supports their development for other diseases driven by non-resolving inflammation. Unwinding the complex molecular machinery controlling SPM production, and dissecting the pro-resolving from the pro-inflammatory pathways triggered by TLR7 may therefore provide a key to the future treatment of a wide range of chronic and devastating diseases by stimulating endogenous resolution mechanisms.

## MATERIALS AND METHODS

### Mice

Wild type and *Alox15*^*−/−*^ mice on a C57BL/6J background were purchased from Jackson Laboratories (USA). Toll-like receptor-7-deficient (*Tlr7*^*−/−*^) mice (Hemmi et al, 2002) were obtained from Prof. S. Akira (Osaka University, Japan) and backcrossed to the C57BL/6J background for at least 10 generations. Mice were fed a normal chow diet containing 18.5% protein and 5.5% fat (Harlan Tekland, Italy) and housed in individually ventilated cages (IVC) under specific pathogen-free conditions in full compliance to FELASA recommendations at the Animal House Facility of the Foundation for Biomedical Research of the Academy of Athens. All procedures had received prior approval from the Institutions and Regional Ethical Review Boards and were in accordance with the US National Institutes of Health Statement of Compliance (Assurance) with Standards for Humane Care and Use of Laboratory Animals (#A5736–01) and with the European Union Directive 86/609/EEC for animal research.

### Experimental protocols and treatments

An established model of allergic airway disease in mice was used (Koltsida et al, 2011; Xirakia et al, 2010). Briefly, 6- to 8-week-old mice were sensitized with two intraperitoneal injections of OVA (Grade V; Sigma) complexed with aluminium hydroxide (alum) and subsequently challenged for 3 consecutive days with aerosolized 5% w/v OVA in PBS (OVA/OVA) for 30 min per day. Control mice received intraperitoneal injections and challenges of PBS (PBS/PBS) or intraperitoneal injections of OVA and challenges of PBS (OVA/PBS). Mice were treated with 200 µg/mouse resiquimod (R-848) or 100 ng/mouse PD1 and/or RvD1 at Day 1 post-allergen challenge as indicated in the experimental protocol. At indicated timepoints, mice were examined for the development of allergic airway inflammation by performing bronchoalveolar lavage, differential cell counting, histological scoring and lymph node T cell assays as detailed below.

### Bronchoalveolar lavage and differential cell counts

BALF of the whole lung was performed twice with 0.5 ml saline. Cells were counted and cell pellets subjected to cytospin centrifugation at 600 rpm for 3 min. Cytospins were stained with May-Grunwald-Giemsa and analyzed by differential cell counting. At all cases, <5 × 10^2^ eosinophils or neutrophils were detectable in BALF from PBS/PBS or OVA/PBS control mice.

### Histological analysis

Lung tissue was fixed in 10% v/v neutral buffered formalin and embedded in paraffin. Paraffin-embedded tissue slices were stained with haematoxylin/eosin (H&E) or periodic acid Schiff's (PAS) solution (Sigma, Deisenhofen, Germany). Histopathologic analysis of inflammatory cells in H&E stained lung sections from at least six mice was performed in a blinded fashion using a semi-quantitative scoring system as previously described (Koltsida et al, 2011; Xirakia et al, 2010). Both peribronchial and perivascular inflammation were scored giving a maximum score of 8 as follows: 0, normal; 1, few cells; 2, a ring of inflammatory cells one cell layer deep; 3, a ring of inflammatory cells two to four cells deep; and 4, a ring of inflammatory cells of more than four cells deep. Histological score for PBS/PBS control mouse lungs was always 0. Morphometric analysis of PAS stained sections was performed by quantifying PAS pixels per µm length distance of bronchial epithelium of central airways using the Image J software. At least six areas from similar sections per mouse and at least six mice were assessed blindly. PAS score for PBS/PBS control mouse lungs was constantly <2.

### Lymph node cultures

Mediastinal lymph nodes were removed, single cell suspensions prepared and cells cultured at 5 × 10^5^/well in 96-well plates in the presence or absence of 100 µg/ml ovalbumin. After 48 h, supernatants were collected and examined by ELISA for the presence of Th1, Th2 and Th17 cytokines. Background levels of cytokine production from PBS/PBS mice were always <20 pg/ml for IL-5, IL-10, IL-13, IL-17 and IFN-γ. DuoSet™ sandwich ELISA kits were from eBioscience (California, USA).

### Airway hyperresponsiveness

Airway hyperresponsiveness was measured in anaesthetized mechanically ventilated mice (flexiVent, SciReq, Montreal, Canada) 24 or 96 h after the last aerosol exposure as previously described (Xirakia et al, 2010).

### Mouse resident macrophage incubations and lipid mediator lipidomics

Peritoneal lavages from 6- to 8-week-old FVB male mice (Charles River Laboratories, Willington, USA) fed with Lab Diet 5001 were harvested, cells left to adhere in 6-well plates and then incubated with 100 µM R-848 (Invivogen, CA, USA), 100 µg/ml Zymosan A (Sigma, MO, USA) or vehicle control (PBS) in the presence of 10 µM d5-DHA (Cayman Chemicals, MI, USA) for 1 h at 37°C. The incubations were stopped using ice-cold methanol and addition of deuterium-labelled standards (d8-5-HETE, d4-PGE_2_ and d4-LTB_4_), and cells/supernatants were collected and kept in −80°C till evaluation. Lipid mediators from these samples were extracted using C-18 solid phase extraction (SPE) and then analyzed using LC-MS–MS. LC-MS-MS-based mediator lipidomics was conducted using linear ion trap quadrupole mass spectrometer (3200 QTRAP, Applied Biosystems, Foster City, CA) equipped with two HPLC pumps (LC20AD, Shimadzu, Columbia, MD) coupled to a reverse phase column (Eclipse Plus C18, 50 mm × 4.6 mm × 1.8 mm, Agilent Technologies Inc., Santa Clara, CA). The mobile phase consisted of methanol:water:acetic acid 60:40:0.01 v/v/v with flow rate at 0.4 ml/min; the mobile phase was gradually increased to 100:0:0.01 over 12 min and kept at 100:0:0.1 for 2 min. All lipid mediators were identified according to published criteria including matching retention times and at least 4–6 prominent diagnostic ions present in their MS/MS spectra (Yang et al, 2011).

### Human monocytes cultures and lipid mediator lipidomics

Peripheral blood mononuclear cells were isolated by dextran-Histopaque (1.077 g/ml) double gradient from whole blood from healthy volunteers (de-identified) who denied taking medications 2 weeks before donation (Partners Human Research Committee Protocol no. 88-02642). Informed consent was obtained from all donors. Peripheral blood monocytes were purified by adherence following plating of PBMCs into 6-well plates (10 × 10^6^/well) and incubation for 1 h at 37°C. Cells were subsequently washed twice with PBS, left to rest for 5 h in RPMI containing 10% FBS, l-glutamine and penicillin/streptomycin, and stimulated for 1 h at 37°C with 100 µM R-848 (Invivogen, CA, USA), 100 µg/ml Zymosan A (Sigma, MO, USA), 100 µM R-848 and 100 mg/ml Zymosan A, or vehicle control (PBS) in the presence of 10 µM d5-DHA (Cayman Chemicals, MI, USA). The experiment was stopped using ice-cold methanol and addition of deuterium-labelled internal standards (d8-5-HETE, d4-PGE2 and d4-LTB4), and samples processed as above.

**The paper explained**

**PROBLEM**

The production of oxygenated lipids derived from polyunsaturated fatty acids, altogether termed specialized proresolving lipid mediators (SPMs), is critical for the resolution of inflammation. Defects or delays in their production result in delayed resolution, prolonged inflammation and increased tissue damage. Still, the mechanisms and pathways that can drive their production, especially in the context of inflammatory disease, remain elusive, thus hampering the rational design of novel therapeutics aiming at actively resolving inflammation.

**RESULTS**

We report that TLR7, a major pattern recognition receptor recognizing viral single stranded RNA and damaged self-RNA, drives the biosynthesis of docosahexaenoic acid (DHA)-derived SPMs (termed D-series SPMs) and the resolution of airway inflammation. Using mouse peritoneal macrophages, human peripheral blood monocytes and a murine model of allergic airway inflammation, we demonstrate that TLR7 triggers the production of DHA-derived monohydroxy metabolome markers (d5-7-HDHA, d5-14-HDHA and d5-17-HDHA), the generation of protectin D1 (PD1) and resolvin D1 (RvD1), and the catabasis of Th2-mediated inflammation. We further show that the production of D-series SPMs is key to TLR7 proresolving function, while intranasal administration of synthetic PD1 or RvD1 can be used therapeutically to drive resolution of allergic airway inflammation.

**IMPACT**

Our study uncovers a previously unsuspected mechanism that links innate immunity with the resolution of inflammation. It also reveals a new molecular pathway triggered by TLR7 that drives the production of omega-3 polyunsaturated fatty acid-derived SPMs such as PD1 and RvD1, and suggests that it is possible to harness TLR function to promote resolution of airway inflammation. This broadens our current view of TLR function in health and disease, and has profound implications for the development of novel therapeutics aiming at actively resolving inflammation. In addition, our study proposes a new mode of action of resiquimod, an existing antiviral drug of the imidazoquinoline family, and establishes TLR7 agonists as the first pharmacological agents beyond aspirin and statins that directly induce the lipoxygenase-dependent biosynthetic pathway leading to the production of SPMs. Finally, our study supports the application of TLR7 agonists for the treatment of allergic asthma and respiratory allergies in general, especially during exacerbations, and their development for other diseases driven by non-resolving inflammation.

### Lipid mediator-lipidomics with lung samples

Frozen lung samples were placed into methanol, and organ samples dispersed in cold methanol using a Kontes tissue grinder to gently elute lipid mediators from the tissue, and after addition of internal standard for specific lipid mediators (as above), were taken for extractions using C18 solid phase extraction (SPE). Samples were taken for analysis using LC-MS–MS and published criteria including matching retention times and at least 4–6 prominent diagnostic ions present in their MS/MS spectra (Yang et al, 2011).

### Macrophage cultures and quantitative real-time PCR

Peritoneal and bone marrow-derived macrophages were cultured in DMEM containing 10% foetal calf serum, 100 U/ml penicillin, 100 µg/ml streptomycin and 2 mM l-glutamine 1% penicillin/streptomycin. For bone marrow-derived macrophages, the medium was also supplemented with 20% L929 supernatant as previously described (Harding, 2001). Peritoneal macrophages were stimulated for 1h with 5 µg/ml R-848 (Invivogen, CA, USA) or 1 µg/ml LPS (Sigma, MO, USA) and collected for further analysis. Similarly, bone marrow macrophages were stimulated for 1 h with 5 µg/ml R-848 or 10 ng/ml LPS (Sigma, MO, USA). Total RNA was isolated using the TRIZOL Reagent (Sigma, MO, USA) and cDNA was generated by RT-PCR using the M-MLV reverse transcriptase according to the manufacturer's instructions (Promega, USA). Real-time quantitative PCR was performed with the SYBR® Green ER™ qPCRSuperMix Universal (Invitrogen, UK). Target mRNA levels were expressed relative to GAPDH. Primer sets used were the following: GAPDH sense 5′-CCAGTATGACTCCACTCACG-3′, GAPDH antisense 5′-CTCCTGGAAGATGGTGATGG-3', Alox5 sense 5′-ACATCCTCAAGCAGCACAGAC-3′, Alox5 antisense 5′-GCATCAATACTCAAAGGGAAGCC-3′, Alox15 sense 5′-CTGCCCGCCTGGTATTCC-3′, Alox15 antisense 5′-AATCCGCTTCAAACAGAGTGC-3′.

### Statistical Analysis

Statistical significance of differences was assessed by Student *t*-test for parametric data and by Mann–Whitney U (MWW) test for nonparametric data. Differences were considered significant when *p* < 0.05.

## Author contributions

OK and SK performed experiments and wrote the paper; KP, TV and ADC performed experiments; CT and PS designed experiments and advised on the analysis of data; CNS and EA designed the experiments, analyzed data and wrote the paper.

## For more information

For general information about allergic asthma and allergic diseases: European Academy of Allergy and Clinical Immunology (EAACI):

http://www.eaaci.net/

European Federation of Allergy and Airways Diseases Patients Associations (EFA):

http://www.efanet.org/

For related EU-funded actions on allergy and asthma: Global Allergy and Asthma European Network (GA2LEN):

http://www.ga2len.net/

Post-infectious immune reprogramming and its association with persistence and chronicity of respiratory allergic diseases:

http://www.predicta.eu/

Webpages of the working group of E. Andreakos:

http://www.immunology-brfaa.gr/ and http://www.bioacademy.gr/

Webpage of the working group of C.N.Serhan:

http://etherweb.bwh.harvard.edu/research/overview/serhan.php

## Notes

As a service to our authors and readers, this journal provides supporting information supplied by the authors. Such materials are peer reviewed and may be re-organized for online delivery, but are not copy-edited or typeset. Technical support issues arising from supporting information (other than missing files) should be addressed to the authors.
