# Supplementary material for: Toll-like receptor 7 stimulates production of specialized pro-resolving lipid mediators and promotes resolution of airway inflammation
Source: EMBO Mol Med. 2013 Apr 15;5(5):762–75. doi: 10.1002/emmm.201201891 (PMC3662318; doi:10.1002/emmm.201201891)
Supplement: Supplementary file 1 [file emmm0005-0762-sd1.pdf]

Toll-like receptor 7 stimulates production of specialized pro-resolving lipid mediators and promotes resolution of airway inflammation

Ourania Koltsida, Sergey Karamnov, Katerina Pyrillou, Thad Vickery, Aikaterini-Dimitra Chairakaki, Constantin Tamvakopoulos, Paschalis Sideras, Charles N. Serhan and Evangelos Andreakos

Corresponding author: Evangelos Andreakos, Biomedical Research Foundation, Academy of Athens

Review timeline:

Submission date:	21 August 2012
Editorial Decision:	19 September 2012
Revision received:	18 January 2013
Editorial Decision:	13 February 2013
Revision received:	27 February 2013
Accepted:	01 March 2013

Transaction Report:

(Note: With the exception of the correction of typographical or spelling errors that could be a source of ambiguity, letters and reports are not edited. The original formatting of letters and referee reports may not be reflected in this compilation.)

Editor: Anneke Funk / Céline Carret

1st Editorial Decision 19 September 2012

Thank you for the submission of your manuscript "Toll-like receptor 7 stimulates production of specialized pro-resolving lipid mediators and promotes resolution of airway inflammation" to EMBO Molecular Medicine. We have now heard back from the three referees whom we asked to evaluate your manuscript and they find the topic potentially interesting. However, they also raise concerns on the study, which should be addressed in a major revision of the manuscript.

Importantly, both Reviewer #1 and #3 highlight that mechanistic insight into the connection between TLR7 signaling and DHA utilization should be provided. In addition, Reviewer #1 notes that the TLR7 ligand important in the used experimental setting should be identified.

Given the balance of these evaluations, we feel that we can consider a revision of your manuscript if you can convincingly address the issues that have been raised within the time constraints outlined below.

Revised manuscripts should be submitted within three months of a request for revision. They will otherwise be treated as new submissions, unless arranged differently with the editor.

I look forward to seeing a revised form of your manuscript as soon as possible.

***** Reviewer's comments *****

Referee #1 (Comments on Novelty/Model System):

The authors provided an insight into molecular mechanism of TLR7-dependent resolution of inflammation. Thus this study is novel and important. However I think that an explanation of the model system was not enough. The authors should identify a ligand for TLR7 to strengthen the adequacy of their experimental system and the reliability of their conclusion. Identification of TLR7-stimulating self-RNA produced from the damaged tissues/cells gives strong evidence for a physiological relevance of TLR7-SPMs-dependent resolution of allergic airway inflammation.

Referee #1 (General Remarks):

Recent studies have shown that lipid mediators regulate a resolution of inflammation as well as an induction of inflammation. Andreakos and colleagues found that TLR7, a receptor recognizing RNA, stimulates production of specialized pro-resolving mediators, PD1 and RvD1, to suppress Th2-type inflammation. The manuscript was well written and the experiments were well controlled. The finding about TLR7-mediated resolution of allergic airway inflammation was interesting. I think this study makes a large impact on the related immunology field, if following issues are clarified: (1) Self-RNAs, such as U1 RNA and micro RNA, released from damaged tissues/cells are capable of stimulating TLR7. What type of self-RNA stimulates TLR7 under this experimental condition? (2) How does TLR7 stimulation enhance DHA utilization and biosynthesis of SPMs?

Referee #2:

This report describes toll-like receptor 7 stimulation of the production of lipid mediators promoting resolution of airway inflammation. This is clearly written and the information contained in the manuscript is novel. It is has potentially far reaching implications in that TLR 7 agonists are in preclinical and clinical development for the therapy of allergic diseases as well as for virus infections and if they have properties that hasten resolution of inflammation as well, then this is an important finding. I have only minor comments for revision.

As the conclusions are well supported by the data, specific comments:

Abstract: please define DHA.

Abstract and throughout the manuscript: please replace "murine" with "mouse" as "murine" pertains to members of the Muridae, which is a family of rodents which includes both mice and rats.

Introduction, page 3: please replace "Celebrex" which is the trade name with the generic name "celecoxib" and do not capitalise. Similarly, do no capitalise "Zileuton".

The Introduction is rather long and the paragraph spanning in pages 3 and 4 in particular could be shortened as in my view there is no need to discuss drugs used to treat asthma. Please keep the focus on resolution of inflammation.

Results
Page 5 and Figure 1: the text and figure should include data relating the resolution of inflammation to cell counts in naive mice, as without this information, we cannot tell whether complete resolution has occurred. Please correct.

Page 7: please define HDHA at first usage.

Page 14: was Ethics approval obtained for the donation of the peripheral blood mononuclear cells by these healthy volunteers? If obtained, please state so and name the Committee approving the approval for the study. If it was not obtained, then we need a clear statement why Ethics approval was not needed for donation of these samples.

Page 17: please provide the full details for the reference from "Cytos".

Figure 1: please split the horizontal axis for Figure 1B and provide a right-hand axis on a different scale so that we can more easily see the differences between groups for mono science neutricals and lipocites.

The same could be done for Figure 2C, 5B and 6B.

Referee #3 (Comments on Novelty/Model System):

Resolution of inflammation is an active process, and treatment of inflammatory diseases based on endogenous agonists of resolution is of wide interest. This is a novel and surprising finding that TLR7 signals promote the resolution of allergic airway inflammation via 12/15-LOX-derived biosynthetic pathways of specialized pro-resolving lipid mediators. Their use of allergic airway model after discontinuation of allergen challenge is quite adequate to address the molecular pathways that regulate the resolution of inflammation.

Referee #3 (General Remarks):

This manuscript addresses interesting questions regarding the role of lipid mediators, especially protectin D1 (PD1) and resolvin D1 (RvD1), as pro-resolving mediators in spatiotemporal resolution of allergic airway inflammation in mice after discontinuation of allergen challenge. The authors clearly demonstrated that administration of TLR7 agonist R-848 exhibited accelerated resolution, and mobilizes the 12/15-LOX-derived biosynthetic pathways that drive the production of PD1 and other pro-resolving mediators. Endogenous production of 12/15-LOX-derived mediators appear to be critical for TLR7-mediated resolution of allergic airway inflammation, since R-848-mediated effect was largely lost in mice deficient in 12/15-LOX. The experiments appear to be well executed, and the results are compelling. There are several points the authors should address.

1. In the previous paper (AJRCCM 181, 1207-1206, 2010), the authors reported that intranasal administration of TLR7 ligand confered suppression of allergic airway inflammation through the induction of type 1 interferons and IFN-gamma-producing CD8+ cells. It is valuable to attempt to correlate the findings here to those in the previous paper, i.e. 12/15-LOX-derived mediators, type 1 interferons and IFN-gamma-producing CD8+ cells in the resolution of allergic airway inflammation.

2. Figure 2, 3: Which cell types are possible targets for TLR7 agonist R-848 treatment in the lung? In which cell types is TLR7 expressed?

3. Figure 4: How TLR7 signals enhance DHA utilization and biosynthesis of 5-LOX and/or 12/15-LOX-derived mediators in macrophages? Which intracellular signals are involved? Is it selective to TLR7 compared to other TLRs?

4. Table S1: Earlier reports have shown that lipoxin A4 are present and can regulate allergic airway responses. Therefore it is surprising that LXA4 was below the detection limits in this experimental setting. Could you comment on this?

1st Revision - authors' response 18 January 2013

<u>*EDITOR*</u>

COMMENT: We have now heard back from the three referees whom we asked to evaluate your manuscript and they find the topic potentially interesting. However, they also raise concerns on the study, which should be addressed in a major revision of the manuscript.

Importantly, both Reviewer #1 and #3 highlight that mechanistic insight into the connection between TLR7 signaling and DHA utilization should be provided. In addition, Reviewer #1 notes that the

TLR7 ligand important in the used experimental setting should be identified.

Given the balance of these evaluations, we feel that we can consider a revision of your manuscript if you can convincingly address the issues that have been raised within the time constraints outlined below.

REPLY: We would like to thank the Reviewers and Editor for their positive comments and insightful suggestions. We have now carefully addressed all Reviewers' comments (Point-to-point Reply below). Importantly, we have added new data on the mechanistic link between TLR7 signaling and SPM biosynthesis (new Fig. 5). We show that TLR7 signaling up-regulates the expression of 5-lipoxygenase and 12/15-lipoxygenase enzymes, rate-limiting components in the metabolism of DHA to its bioactive derivatives. We have also added a new Figure in the Supporting information (Fig S5) revealing the presence of extracellular RNA aggregates in inflamed tissue. This is presumably released from damaged cells and potentially constitutes an endogenous ligand for TLR7 *in vivo*.

REVIEWERS

Referee #1 (Comments on Novelty/Model System):

The authors provided an insight into molecular mechanism of TLR7-dependent resolution of inflammation. Thus this study is novel and important. However I think that an explanation of the model system was not enough. The authors should identify a ligand for TLR7 to strengthen the adequacy of their experimental system and the reliability of their conclusion. Identification of TLR7-stimulating self-RNA produced from the damaged tissues/cells gives strong evidence for a physiological relevance of TLR7-SPMs-dependent resolution of allergic airway inflammation.

REPLY: We thank the reviewer for his positive remarks and suggestions. We have addressed these concerns as detailed below.

Referee #1 ((General Remarks):

Recent studies have shown that lipid mediators regulate a resolution of inflammation as well as an induction of inflammation. Andreakos and colleagues found that TLR7, a receptor recognizing RNA, stimulates production of specialized pro-resolving mediators, PD1 and RvD1, to suppress Th2-type inflammation. The manuscript was well written and the experiments were well controlled. The finding about TLR7-mediated resolution of allergic airway inflammation was interesting. I think this study makes a large impact on the related immunology field, if following issues are clarified:

(1) Self-RNAs, such as U1 RNA and micro RNA, released from damaged tissues/cells are capable of stimulating TLR7. What type of self-RNA stimulates TLR7 under this experimental condition?

REPLY: It would certainly be very useful to know the identity of self-RNAs that are capable of stimulating TLR7 *in vivo*. Damaged RNA often aggregated and complexed with autoantibodies or cationic peptides, uridine-rich RNA and microRNAs have all been proposed to constitute endogenous ligands for TLR7 (Barrat FJ et al. JEM 2005; Ganguli D. et al. JEM 2008; Diebold SS et al. EJI 2006; Barbalat R et al. Ann Rev Immunol 2011; Fabbri M. et al. PNAS 2012; Green NM et al. JBC 2012). However, their detection at the level of the tissue and their distinction from non-stimulatory RNA is very difficult, while their immune-stimulatory capacity is dependent on their extracellular localization and their eventual access to the endosomal compartments. We have therefore tested carefully for the presence of extracellular RNA in inflamed lung using an established protocol (Ganguli D. et al. JEM 2008; Salagianni M. et al. Circulation 2012) and found that RNA aggregates-presumably released from damaged cells- are detectable in a cellular areas of the inflamed tissue. These are likely to constitute endogenous ligands for TLR7 and contribute to TLR7-mediated signaling *in vivo* (new Fig S5 in Supporting Information). A comment on this point and possibility has now been added to the Discussion (p.12).

(2) How does TLR7 stimulation enhance DHA utilization and biosynthesis of SPMs?

REPLY: The capacity of TLR7 to enhance the biosynthesis of D-series SPMs is possibly the result, in part, of its ability to up-regulate the expression of lipoxygenase enzymes. We found that in cultured peritoneal and bone marrow-derived macrophages, TLR7 stimulation rapidly increases 5-lipoxygenase and 12/15-lipoxygenase levels (new Fig 5), rate-limiting components in the metabolism of DHA to its bioactive mediators (Hong S et al. JBC 2003; Serhan CN et al. JI 2006; Miyata J et al. JACI 2012). Notably, 12/15-lipoxygenase expression has been recently shown to mark alternatively activated macrophages and to play a key role in the clearance of apoptotic cells and the maintenance of tissue homeostasis (Uderhardt S et al. Immunity 2012), raising the possibility that TLR7 stimulation, alternatively activated macrophage function and DHA-derived SPM production are all linked. However, TLR7-mediated regulation of lipoxygenase expression may not be the only mechanism involved. It is well established that substrate availability is another critical factor that determines the biosynthetic rate of SPMs. *In vivo*, the sources for substrate are multiple and include resident cells that have esterified DHA in membrane phospholipids (such as phosphatidylethanolamine and phosphatidylcholine), infiltrating leukocytes that also carry esterified DHA, and oedema itself that brings into the inflamed site unesterified DHA (Hong S et al. JBC 2003; Marcheselli VL et al. JBC 2003; Kasuga K et al. JI 2008). The later appears to be the dominant source of substrate, providing at least 50% of the DHA needed for the generation of SPMs in animal models of acute inflammation (Kasuga K et al. JI 2008; Serhan CN AJP 2010). It is therefore possible that TLR7 also affects substrate availability as it has been previously shown that the TLR7/8 signaling pathway (i) has the capacity to mobilize eicosanoids from esterified lipids in a cytosolic (calcium-dependent and independent) phosphilipase A2-dependent manner, and (ii) causes vasodilation and enhanced oedema formation (Hattermann K, Picard et al. FASEB J 2007). We have now added these new data (new Fig.5) and commented on these possibilities in the Discussion (p.11).

Referee #2:

This report describes toll-like receptor 7 stimulation of the production of lipid mediators promoting resolution of airway inflammation. This is clearly written and the information contained in the manuscript is novel. It is has potentially far reaching implications in that TLR 7 agonists are in preclinical and clinical development for the therapy of allergic diseases as well as for virus infections and if they have properties that hasten resolution of inflammation as well, then this is an important finding. I have only minor comments for revision.

As the conclusions are well supported by the data, specific comments:

REPLY: We thank the reviewer for his positive remarks and careful reading of the manuscript.

Abstract: please define DHA.

REPLY: DHA stands for docosahexaenoic acid (C22:6). We have now defined that in the text (p.2).

Abstract and throughout the manuscript: please replace "murine" with "mouse" as "murine" pertains to members of the Muridae, which is a family of rodents which includes both mice and rats.

REPLY: We have corrected that throughout the manuscript.

Introduction, page 3: please replace "Celebrex" which is the trade name with the generic name "celecoxib" and do not capitalize. Similarly, do no capitalize "Zileuton".

REPLY: We have corrected that (p.3).

The Introduction is rather long and the paragraph spanning in pages 3 and 4 in particular could be shortened as in my view there is no need to discuss drugs used to treat asthma. Please keep the focus on resolution of inflammation.

REPLY: We have shortened the Introduction (to 3 pages/~710 words long) and deleted the part referring to the drugs used to treat asthma.

Results
Page 5 and Figure 1: the text and figure should include data relating the resolution of inflammation to cell counts in naive mice, as without this information, we can not tell whether complete resolution has occurred. Please correct.

REPLY: We would like to thank the Reviewer for pointing this out. In the BALF of challenged mice inflammatory cells declined to the levels of unchallenged mice by day 10. However, in lung tissue sections resolution is somehow slower. At day 10, there are still significant levels of inflammatory infiltrates and significant presence of goblet cells. We had a comment for that in the original submission, which we have now made clearer in the revised manuscript (p.5). We have also corrected the figure to indicate leukocyte numbers in unchallenged mice (Fig.1).

Page 7: please define HDHA at first usage.

REPLY: HDHA stands for hydroxydocosahexaenoic acid. We have now defined that in the text (p.7).

Page 14: was Ethics approval obtained for the donation of the peripheral blood mononuclear cells by these healthy volunteers? If obtained, please state so and name the Committee approving the approval for the study. If it was not obtained, then we need a clear statement why Ethics approval was not needed for donation of these samples.

REPLY: Ethics approval and informed consent was obtained for all donors (Partners Human Research Committee Protocol n. 88-02642). This is stated on p.16 of the revised manuscript.

Page 17: please provide the full details for the reference from "Cytos".

REPLY: This was an online resource presenting the results of a clinical trial. We have corrected that according to the guidelines of the journal (p.4).

Figure 1: please split the horizontal axis for Figure 1B and provide a right-hand axis on a different scale so that we can more easily see the differences between groups for mono science neutricals and lipocites. The same could be done for Figure 2C, 5B and 6B.

REPLY: We have now corrected that.

Referee #3 *(Comments on Novelty/Model System):*

Resolution of inflammation is an active process, and treatment of inflammatory diseases based on endogenous agonists of resolution is of wide interest. This is a novel and surprising finding that TLR7 signals promote the resolution of allergic airway inflammation via 12/15-LOX-derived biosynthetic pathways of specialized pro-resolving lipid mediators. Their use of allergic airway model after discontinuation of allergen challenge is quite adequate to address the molecular pathways that regulate the resolution of inflammation.

REPLY: We thank the reviewer for his positive remarks.

Referee #3 (General Remarks):

This manuscript addresses interesting questions regarding the role of lipid mediators, especially protectin D1 (PD1) and resolvin D1 (RvD1), as pro-resolving mediators in spatiotemporal resolution of allergic airway inflammation in mice after discontinuation of allergen challenge. The authors clearly demonstrated that administration of TLR7 agonist R-848 exhibited accelerated resolution, and mobilizes the 12/15-LOX-derived biosynthetic pathways that drive the production of

© EMBO

PD1 and other pro-resolving mediators. Endogenous production of 12/15-LOX-derived mediators appear to be critical for TLR7-mediated resolution of allergic airway inflammation, since R-848-mediated effect was largely lost in mice deficient in 12/15-LOX. The experiments appear to be well executed, and the results are compelling. There are several points the authors should address.

1. In the previous paper (AJRCCM 181, 1207-1206, 2010), the authors reported that intranasal administration of TLR7 ligand conferred suppression of allergic airway inflammation through the induction of type 1 interferons and IFN-gamma-producing CD8+ cells. It is valuable to attempt to correlate the findings here to those in the previous paper, i.e. 12/15-LOX-derived mediators, type 1 interferons and IFN-gamma-producing CD8+ cells in the resolution of allergic airway inflammation.

REPLY: Our previous study evaluated the anti-inflammatory/ immunomodulatory role of TLR7 in allergic airway inflammation. The TLR7 agonist was administered before allergen challenge and disease was evaluated at day 1 post-challenge, the peak of the inflammatory response. On the contrary, our present study evaluated the role of TLR7 in the resolution of allergic airway inflammation. The TLR7 agonist was administered at day 1 post-challenge, in already inflamed lung, and its effects in the resolution of inflammation were assessed in a spatiotemporal manner. Although the two studies address the role of TLR7 at distinct phases of the inflammatory response, and thus are not directly comparable, interesting implications arise.

It becomes apparent that TLR7 acts at multiple levels to suppress allergic airway disease. Through the induction of type I IFNs, TLR7 rapidly shuts down Th2-mediated pro-inflammatory responses in the airways. Through the production of D-series SPMs, TLR7 promotes resolution of allergic airway inflammation. Finally, through the generation of regulatory T cell populations secreting IFNγ, TLR7 ensures long-term suppression of allergic responses and long-term remission of allergic airway disease.

Notably, type I IFNs and 12/15-lipoxygenase promote the expression of IFNγ (Zhao L et al. JBC 2002; Middleton MK et al. JI 2006; Dioszeghy et al. JI 2008; Middleton MK et al. Infect Immun 2009; Haworth et al. Nat Med 2008), while IFNγ acts as a negative feedback mechanism to down-regulate the expression of 12/15-lipoxygenase and the generation of 12/15-lipoxygenase-derived products (Conrad DJ et al. PNAS 1992; Nassar GM et al. JBC 1994). This suggests the existence of a complex molecular network triggered by TLR7 that aims at resolving inflammation and restoring tissue homeostasis.

These possibilities are now discussed (p.9-10).

2. Figure 2, 3: Which cell types are possible targets for TLR7 agonist R-848 treatment in the lung? In which cell types is TLR7 expressed?

REPLY: TLR7 is abundantly expressed in the lung. Lung macrophages, myeloid dendritic cells and plasmacytoid dendritic cells all express abundant TLR7 levels and respond to TLR7 stimulation (Maris et al. ERJ 2006; Demedts IK et al. AJRCMB 2006; Bessa J et al. JI 2009; Desch N. JEM 2011). Bronchial epithelial cells also express high levels of TLR7 (Xirakia et al. AJRCCM 2010; Cherfils-Vicini J et al. JCI 2010) as do eosinophils that infiltrate the lung during inflammation (Nagase H et al. JI 2003). In contrast, alveolar epithelial cells and neutrophils lack significant TLR7 expression (Janke M et al. JACI 2009; Xirakia et al. AJRCCM 2010). Therefore, the cellular targets for the TLR7 agonist R-848 are multiple, and the effects of TLR7 stimulation complex. A comment for that has now been included in the Discussion (p.12).

3. Figure 4: How TLR7 signals enhance DHA utilization and biosynthesis of 5-LOX and/or 12/15-LOX-derived mediators in macrophages? Which intracellular signals are involved? Is it selective to TLR7 compared to other TLRs?

REPLY: The capacity of TLR7 to enhance the biosynthesis of D-series SPMs is likely the result of several components including its ability to up-regulate the expression of lipoxygenase enzymes. We found that in cultured peritoneal and bone marrow-derived macrophages, TLR7 stimulation rapidly increases 5-lipoxygenase and 12/15-lipoxygenase levels (new Fig 5), rate-limiting components in the metabolism of DHA to its bioactive derivatives (Hong S et al. JBC 2003; Serhan CN et al. JI

2006; Miyata J et al. JACI 2012). Notably, 12/15-lipoxygenase expression has been recently shown to mark alternatively activated macrophages and to play a key role in the clearance of apoptotic cells and the maintenance of tissue homeostasis (Uderhardt S et al. Immunity 2012), raising the possibility that TLR7 stimulation, alternatively activated macrophage generation and DHA-derived SPM production are all linked. However, TLR7-mediated regulation of lipoxygenase expression may not be the only mechanism involved. It is well established that substrate availability is another critical factor that determines the biosynthetic rate of SPMs. *In vivo*, the sources for substrate are multiple and include resident cells that have esterified DHA in membrane phospholipids (such as phosphatidylethanolamine and phosphatidylcholine), infiltrating leukocytes that also carry esterified DHA in their membranes, and oedema itself that brings into the inflamed site unestefified DHA from plasma (Hong S et al. JBC 2003; Marcheselli VL et al. JBC 2003; Kasuga K et al. JI 2008). The later appears to be the dominant source of substrate, providing at least 50% of the DHA needed for the generation of SPMs in animal models of acute inflammation (Kasuga K et al. JI 2008; Serhan CN AJP 2010). It is therefore possible that TLR7 also affects substrate availability as it has been previously shown that the TLR7/8 signaling pathway (i) has the capacity to mobilize eicosanoids from esterified lipids in a cytosolic (calcium-dependent and independent) phosphilipase A2-dependent manner, and (ii) causes vasodilation and enhanced oedema formation (Hattermann K et al. FASEB J 2007). We have now added these new data as new Fig 5 and commented on these points within the revised Discussion (p.11).

An interesting question that arises is whether other TLRs can also affect the biosynthesis of D-series mediators. We found that the up-regulation of both 5-lipoxygenase and 12/15-lipoxygenase expression was selective for TLR7; LPS did not up-regulate any of these enzymes. However, this does not exclude a role for other TLRs in SPM biosynthesis. In addition, the effect of TLRs on substrate availability has not been addressed. As several TLRs have been shown to activate cytosolic PLA2 enzymes that induce the production of eicosanoids (Qi HY et al. JBC 2005; Norris PC and Dennis EA PNAS 2012), and to enhance the formation of oedema (Alvarez Y et al. Mediators Inflam 2010; Takeuchi O and Akira S Cell 2010), it is tempting to speculate that other TLR family members may also be capable of promoting the generation of SPMs and the resolution of inflammation. We have now added these new data (new Fig 5) and commented on these possibilities in the Discussion (p.11).

4. Table S1: Earlier reports have shown that lipoxin A4 are present and can regulate allergic airway responses. Therefore it is surprising that LXA4 was below the detection limits in this experimental setting. Could you comment on this?

REPLY: This could be due to differences in the mouse strain used, the biological material analyzed and/or the diet. In the study of Levy et al. Nat Med 2002, BALB/c were used and lipoxin A4 levels were quantified in the BAL fluid. In contrast, in the present study C57BL/6 mice were employed and SPMs were quantified in total lung homogenates. BALF fluid was not examined. Differences in the diet could further affect the generation of lipoxin A4 in these two different experimental settings. We have now included a comment for that in the Discussion (p.11).

2nd Editorial Decision 13 February 2013

Thank you for the submission of your revised manuscript to EMBO Molecular Medicine. We have now received the enclosed reports from the referees that were asked to re-assess it. As you will see the reviewers are now supportive and I am pleased to inform you that we will be able to accept your manuscript pending editorial final amendments.

***** Reviewer's comments *****

Referee #1 (General Remarks):

The authors addressed my concerns and the manuscript was considerably improved.

Referee #3 (General Remarks):

The revised manuscript takes into account each of the points raised by reviewers and is substantially improved. Additional experiments were performed and new results that further strengthen the author's conclusion are given. These are novel and potential important new results with implications given the role of TLR7-Alox15 axis as an endogenous pro-resolution program in the airway inflammation.
