# Supplementary material for: Toll-like receptor 7 stimulates production of specialized pro-resolving lipid mediators and promotes resolution of airway inflammation
Source: EMBO Mol Med. 2013 Apr 15;5(5):762–75. doi: 10.1002/emmm.201201891 (PMC3662318; doi:10.1002/emmm.201201891)
Supplement: Supplementary file 2 [file emmm0005-0762-sd2.pdf]

Supporting Information

Toll-like receptor 7 stimulates production of specialized pro-resolving lipid mediators and promotes resolution of airway inflammation

Ourania Koltsida, Sergey Karamnov, Katerina Pyrillou, Thad Vickery, Aikaterini-Dimitra Chairakaki[1], Constantin Tamvakopoulos, Paschalis Sideras, Charles N. Serhan and Evangelos Andreakos

TABLE OF CONTENTS

**Table S1**	Lipid mediator profiles in the lung of OVA sensitized and challenged mice treated with R-848 or vehicle control (PBS) on day 1 post-challenge	2
**Figure S1**	Airway hyper-responsiveness during resolution of allergic airway inflammation	3
**Figure S2**	TLR7 is essential for R-848-mediated resolution of allergic airway inflammation	4
**Figure S3**	Production of eicosanoids LTB4, PGE2, PGD2 and PGF2a during the development and resolution of allergic airway inflammation	5
**Figure S4**	R-848 stimulates production of d5-DHA-derived specialized lipid mediator precursors by human peripheral blood monocytes	6
**Figure S5**	Extracellular RNA aggregates are detectable in acellular areas of the inflamed tissue	7

SUPPORTING INFORMATION TABLES

Table S1. Lipid mediator profiles in the lung of OVA sensitized and challenged mice treated with R-848 or vehicle control (PBS) on day 1 post-challenge. Data are expressed as pg/mg tissue. Q1: Precursor ion; Q3: Target specific product ion; BL: Below limits

Compound	*Q1*	*Q3*	Day -2	Day 1	Day 2		Day 4		Day 7		Day 10	
			-	-	Vehicle	R-848	Vehicle	R-848	Vehicle	R-848	Vehicle	R-848
5-HETE	319	115	3.21	6.47	3.59	8.41	7.66	3.02	6.15	4.67	3.52	3.42
12-HETE	319	179	32.17	100.65	64.87	117.84	150.09	91.11	122.88	103.55	60.24	49.46
15-HETE	319	219	9.80	32.74	24.66	43.54	53.68	37.60	49.61	35.47	19.38	21.27
7-HDHA	343	141	5.89	0.30	0.05	0.09	0.07	0.06	0.06	0.08	0.12	0.23
14-HDHA	343	205	14.47	45.22	34.71	76.79	68.75	51.57	62.46	46.40	23.50	21.17
17-HDHA	343	245	6.85	23.78	12.00	26.91	30.04	24.09	26.33	19.44	10.38	10.75
PD1	359	153	0.76	3.37	3.35	10.84	6.97	5.29	6.22	5.52	1.38	1.51
RvD1	375	215	BL	BL	BL	BL	BL	BL	BL	BL	BL	BL
RvD2	375	175	BL	BL	BL	BL	BL	BL	BL	BL	BL	BL
PGE_2	351	189	143.04	193.23	184.37	353.84	311.41	164.99	390.53	315.84	164.41	282.51
PGD_2	351	233	13.08	9.11	16.65	39.79	27.74	32.92	30.09	36.14	12.60	25.44
PGF2α	353	193	11.63	6.16	7.82	10.08	11.15	5.82	13.30	9.90	7.81	10.41
LTB_4	335	195	1.03	1.34	1.30	3.18	2.80	1.22	1.94	1.86	0.77	0.72
20-OH-LTB_4	351	195	BL	BL	BL	BL	BL	BL	BL	BL	BL	BL
LXA_4	351	115	BL	BL	BL	BL	BL	BL	BL	BL	BL	BL
LXB_4	351	217	BL	BL	BL	BL	BL	BL	BL	BL	BL	BL
18-HEPE	317	259	0.72	0.40	0.22	0.40	0.25	0.24	0.38	0.27	0.45	0.45
RvE1	349	195	BL	BL	BL	BL	BL	BL	BL	BL	BL	BL

SUPPORTING INFORMATION FIGURES

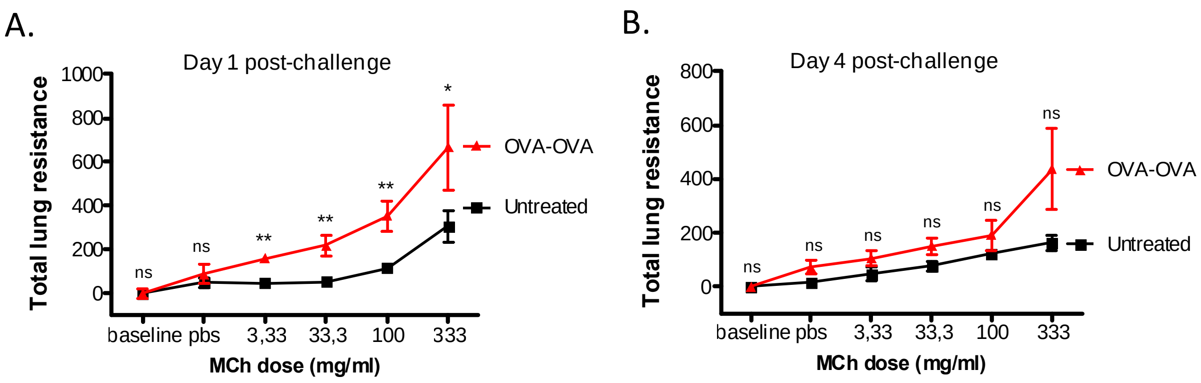

Figure S1. Airway hyper-responsiveness during resolution of allergic airway inflammation.

Airway hyper-responsiveness of OVA sensitized and challenged miceor untreated controls at day 1 and day 4 post-OVA challenge. Airway hyper-responsiveness was measured as methacholine-induced increases in total lung resistance (RL) in mechanically ventilated mice. Data are expressed as mean values of percentage increase from baseline of the total RL ± SEM of 5-7 mice per group from two independent experiments.

*p< 0.05, **p< 0.01, ns: non-significant compared to untreated control

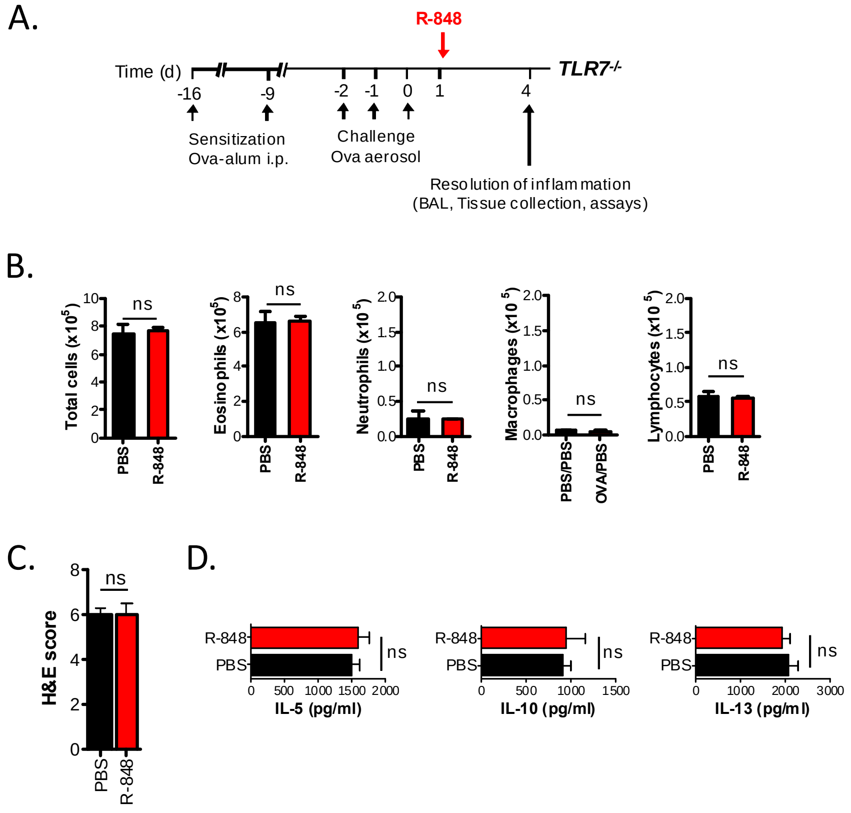

Figure S2. TLR7 is essential for R-848-mediated resolution of allergic airway inflammation.

A. Protocol of R-848 (200 µg/mouse) or vehicle (PBS) administration in *Tlr7*$^{-/-}$ mice. **B.** Total and differential cell counts in BALF of OVA sensitized and challenged (OVA/OVA) mice at day 4 post-challenge. Results are expressed as mean ± SEM of 5-6 mice per group. **C.** Histological assessment of lung inflammation of *Tlr7*$^{-/-}$ mice at day 4 post-challenge. Hematoxylin and eosin (H&E)-stained lung sections and histological scoring expressed as mean values ± SEM from 5-6 mice/group are shown. **D.** Allergen-specific effector T cell responses in mediastinal LNs of *Tlr7*$^{-/-}$ mice at day 4 post-challenge. Cytokine levels are expressed as mean values ± SEM in supernatants of OVA-stimulated mediastinal LN cultures of 5-6 mice per group.

ns: non-significant compared to vehicle-treated control

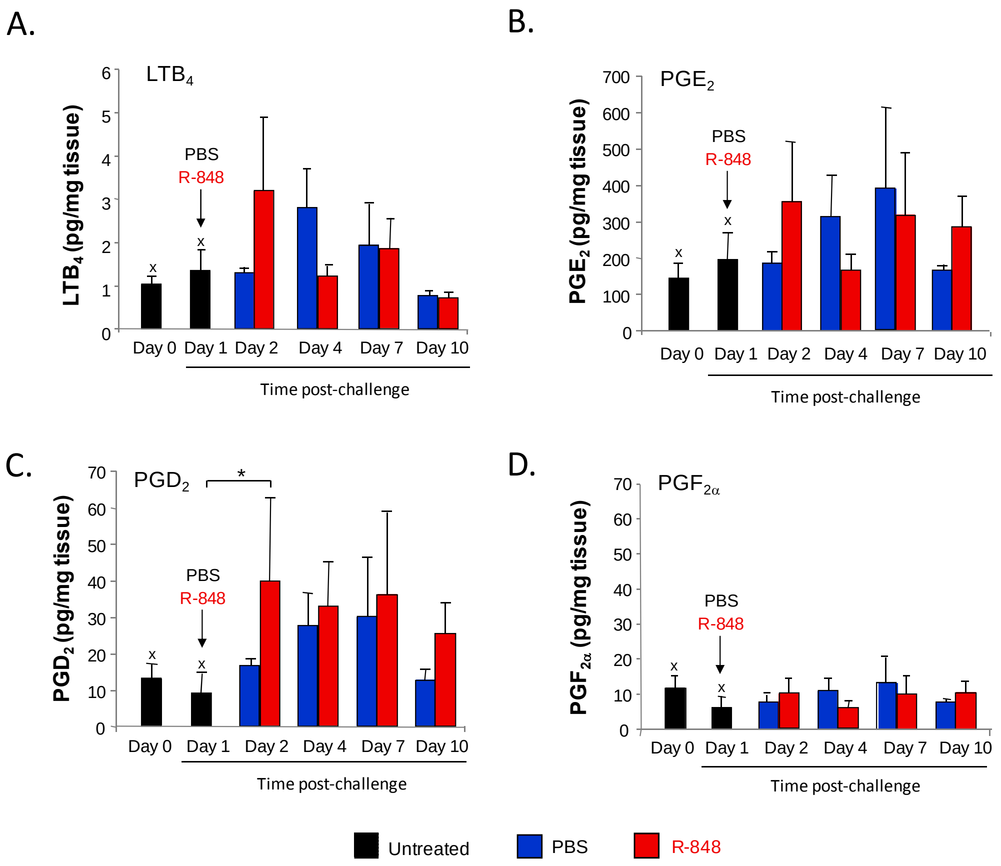

Figure S3. Production of eicosanoids LTB_4, PGE_2, PGD_2 and PGF_{2a} during the development and resolution of allergic airway inflammation.

OVA sensitized and challenged mice treated with 200 µg R-848 or vehicle control (PBS) on day 1 post-challenge were sacrificed at target time points and lungs were harvested and homogenized. Lipid mediators were extracted with deuterium-labeled internal standards (d4-PGE2, d8-5SHETE and d4-LTB4 from Cayman Chemical) using C-18 solid phase extraction. **A-D.** Time course of LTB_4 (**A**), PGE_2 (**B**), PGD_2 (**C**) and PGF_{2a} (**D**) lipid mediators produced in murine lungs. Black bars indicate baseline levels before administration of R-848. Black bars indicate vehicle. Blue bars indicate treatment with R-848. Results are expressed as mean ± SEM of n=4-5 mice per group. $^{*}p<0.05$; $^{X}p<0.05$ compared to 0.

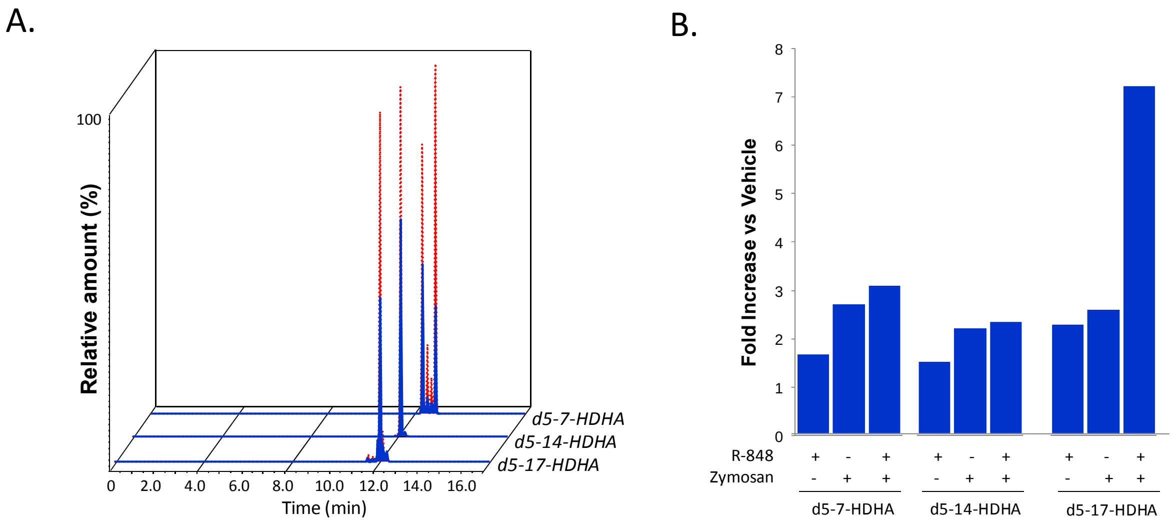

Figure S4. R-848 stimulates production of d5-DHA-derived specialized lipid mediator precursors by human peripheral blood monocytes.

Monocytes obtained from peripheral blood of healthy human donors were cultured in PBS containing 100 µM R-848 (Invivogen, CA, USA), 100 mg/ml Zymosan A (Sigma, MO, USA), 100 µM R-848 and 100 mg/ml Zymosan A, or vehicle control (PBS) in the presence of 10 µM d5-DHA (Cayman Chemicals, MI, USA). After 1 hour at 37^0C, supernatants were collected and lipid mediators extracted with deuterium-labeled internal standards (d4-PGE2, d8-5SHETE and d4-LTB4 from Cayman Chemical) using C-18 solid phase extraction. **A.** LC-MS-MS chromatograms of SPM precursors, d5-7-HDHA, d5-14-HDHA and d5-17-HDHA produced by untreated (solid blue line) and R-848-treated (dashed red line) human monocytes. **B.** Quantitation of SPM precursors. Results are representative of n=3 independent experiments.

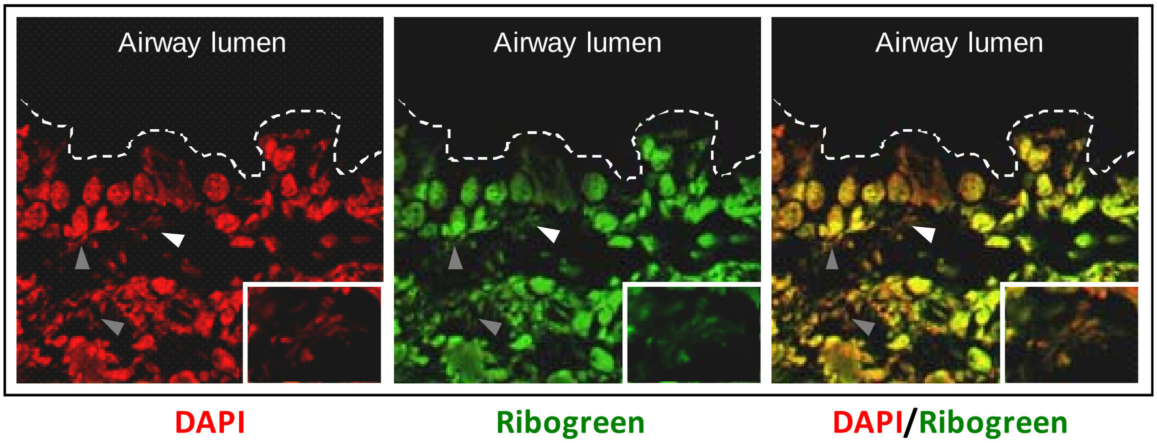

Figure S5. Extracellular RNA complexes are present in inflamed lung

C57BL/6 mice were sensitized and challenged by ovalbumin, and analyzed at day 1 post-challenge for the presence of extracellular RNA in acellular areas of the inflamed tissue. Representative fluorescent photomicrographs of Ribogreen (green) and DAPI (pseudocoloured red)-stained sections from the aortic root are shown (original magnification 120X). Colocalized signal appears yellow. Arrowheads indicate extracellular RNA aggregates. Inserts (bottom right of each picture) show high magnification images of areas marked with white arrowheads.

.
